# *Streptococcus dysgalactiae* subsp. *equisimilis* Isolated From Infections in Dogs and Humans: Are Current Subspecies Identification Criteria accurate?

**DOI:** 10.1007/s00284-016-1113-x

**Published:** 2016-08-09

**Authors:** Marcin Ciszewski, Kamil Zegarski, Eligia M. Szewczyk

**Affiliations:** Department of Pharmaceutical Microbiology and Microbiological Diagnostics, Medical University of Łódź, 137 Pomorska St., 90-235 Łódź, Poland

## Abstract

*Streptococcus dysgalactiae* is a pyogenic species pathogenic both for humans and animals. Until recently, it has been considered an exclusive animal pathogen causing infections in wild as well as domestic animals. Currently, human infections are being reported with increasing frequency, and their clinical picture is often similar to the ones caused by *Streptococcus pyogenes*. Due to the fact that *S. dysgalactiae* is a heterogeneous species, it was divided into two subspecies: *S. dysgalactiae* subsp. *equisimilis* (SDSE) and *S. dysgalactiae* subsp. *dysgalactiae* (SDSD). The first differentiation criterion, described in 1996, was based on strain isolation source. Currently applied criteria, published in 1998, are based on hemolysis type and Lancefield group classification. In this study, we compared subspecies identification results for 36 strains isolated from clinical cases both in humans and animals. Species differentiation was based on two previously described criteria as well as MALDI-TOF and genetic analyses: RISA and 16S rRNA genes sequencing. Antimicrobial susceptibility profiles were also determined according to CLSI guidelines. The results presented in our study suggest that the subspecies differentiation criteria previously described in the above two literature positions seem to be inaccurate in analyzed group of strains, the hemolysis type on blood agar, and Lancefield classification should not be here longer considered as criteria in subspecies identification. The antimicrobial susceptibility tests indicate emerging of multiresistant human SDSE strains resistant also to vancomycin, linezolid and tigecycline, which might pose a substantial problem in treatment.

## Introduction

*Streptococcus dysgalactiae* is a pyogenic species pathogenic both for humans and animals. Until recently, it has been considered an exclusive animal pathogen causing infections in wild animals, livestock, and pets. Currently, human infections are being reported with increasing frequency. Their clinical picture is often similar to the ones caused by group A streptococcus (*Streptococcus pyogenes*) [3].

*S. dysgalactiae* proved to be a heterogeneous species, what encouraged researchers to divide it into two subspecies: *S. dysgalactiae* subsp. *equisimilis* (SDSE) and *S. dysgalactiae* subsp. *dysgalactiae* (SDSD). The first differentiation criterion was proposed in 1996 by Vandamme et al. [22], second criteria—only 2 years later—in 1998 by Vieira et al. [23] which are being applied up to the present. Over the years, controversies arose over this classification, showing that the streptococci taxonomy has not been completed yet and generally used *S. dysgalactiae* subspecies division causes more questions than solutions. In some research reports, subspecies names are used instead of species (i.e., *S. equisimilis*), which causes unnecessary confusion in diagnostics [4, 19].

The presented study provides a new view on *S. dysgalactiae* identification, subspecies differentiation criteria and similarities between strains isolated from human and animal clinical materials.

## Materials and Methods

### Bacterial Strains

30 human *S. dysgalactiae* isolates from clinical cases (mainly wounds, abscesses, bedsores, noses, ears, throats) were obtained from Synevo Medical Laboratory in Łódź, Poland.

6 animal *S. dysgalactiae* isolates from clinical cases in pets (dogs—from wounds, abscesses, birth canal) were obtained from VETCOMPLEX Veterinary Diagnostic Centre in Łódź, Poland.

### Phenotypic Bacterial Identification According to Vieira et al. [23]

Hemolysis type was evaluated as well as Lancefield group. The type of hemolysis was observed after a 24-h incubation at 37 °C (aerobic atmosphere) on agar plates containing 5 % defibrinated sheep blood. The type of surface C-polysaccharide, classifying analyzed strains to serological groups according to Lancefield, was evaluated using latex agglutination tests with Prolex Streptococcal Grouping Latex Kit.

### Bacterial Identification Based on Cell Proteins Structure and DNA Sequences

MALDI-TOF technique (matrix-assisted laser desorption ionization-time of flight) [5] which compares cell proteins specters with database (bioMérieux VITEK^®^ MS) was employed to identify the analyzed clinical strains.

All bacterial strains were also identified by means of RISA (16S–23S rDNA intergenic spacer region) method. PCR reactions were conducted using *S. dysgalactiae* species-specific primers (Fwd 5′-TGGAACACGTTAGGGTCG-3′, Rev 5′-CTTAACTAGAAAAACTCTTGATTATTC-3′) [9] and SDSE subspecies-specific primers (Fwd 5′-GGTACTGTTGAGGGGACGAA-3′, Rev 5′-CGATTGAGGAGTCACGTTCA-3′) [16].

Genetic identification of the selected strains was also confirmed by means of amplification of 16S rRNA coding genes from the selected strains, using primers described in the paper (8F 5′-GAGAGTTTGATCCTGGCTCAG-3′, 1942R 5′-TACGGCTACCTTGTTACGACT-3′) [21]. Afterward, amplified DNA fragments were sequenced and compared with Genbank database. The obtained sequences were published in Genbank.

### Antimicrobial Susceptibility

The antibiotic susceptibility tests of the analyzed strains were performed by means of disk diffusion method according to Clinical & Laboratory Standards Institute (CLSI) [6] and Polish National Reference Centre for Antibiotics (KORLD) [26] guidelines. Susceptibility to 10 antibiotics, representative of the main antibiotic classes and recommended for streptococcal infections treatment, were determined: penicillin (10 U), erythromycin (15 µg), clindamycin (2 µg), ofloxacin (5 µg), levofloxacin (5 µg), vancomycin (30 µg), tetracycline (30 µg), tigecycline (15 µg), linezolid (30 µg), and cefotaxime (30 µg). The results were interpreted according to CLSI recommendations [6].

## Results

Our findings refer to the strains collected over the period of 2014 in the area of Łódź. They were more frequently isolated from purulent infections in humans than in animals.

The analyzed *S. dysgalactiae* strains were assigned to subspecies according to the criteria proposed by Vandamme et al., Vieira et al., as well as to the results of MALDI-TOF method and genetic studies (Table 1). The classification by Vandamme et al. was based on strain isolation sources. 30 human isolates were classified as SDSE, whereas 6 animal isolates were classified as SDSD subspecies.

**Table 1:** The comparison of *S. dysgalactiae* subspecies identification results according to various criteria

Groups of strains	Hemolysis type	Lancefield group	Subspecies according to Vandamme et al. [22]	Subspecies according to Vieira et al. [23]	Subspecies according to MALDI-TOF and genetic criteria
Human *S. dysgalactiae* isolates					
SV106, SV260, SV376, **SV430**, **SV468**, SV469, SV515, SV543, SV598, SV650, SV668	β	C	SDSE	SDSE	SDSE
**SV159**, SV296, SV464, SV475, SV540, SV588, SV634, SV652, SV662, **SV773**	β	G	SDSE	SDSE	SDSE
SV204, SV252, **SV279**, SV282, SV301	α	C	SDSE	SDSD	SDSE
SV202, SV213, SV387, **SV631**	α	G	SDSE	—–	SDSE
Animal *S. dysgalactiae* isolates					
**Z2**	β	C	SDSD	SDSE	SDSE
K1, K2, K3, Z6	β	G	SDSD	SDSE	SDSE
Z1	α	G	SDSD	—–	SDSE

Subspecies identification according to Vieira et al. was based on phenotypic characteristics: hemolysis type and Lancefield group. Among 30 human *S. dysgalactiae* isolates, nine (30 %) demonstrated α-type hemolysis, whereas 21 (70 %) demonstrated β-type hemolysis on blood agar. Among animal isolates, one (17 %) showed α-type hemolysis, whereas other five (83 %) showed β-type hemolysis on blood agar. Both human and animal isolates were classified to Lancefield groups C or G. 16 (53 %) human isolates belonged to group C, 14 (47 %) to group G. Among animal isolates, 1 (17 %) was classified to group C, whereas other 5 (83 %) to Lancefield group G. According to subspecies division criteria proposed by Vieira et al., among human isolates, 21 (70 %) might be classified to SDSE, 5 (17 %) to SDSD, but 4 isolates (13 %) did not match the criteria for neither of the subspecies. 5 (83 %) animal isolates might be classified to SDSE, whereas 1 (17 %) did not match the criteria for neither of the subspecies according to Vieira et al.

All analyzed strains were unequivocally identified as SDSE by means of MALDI-TOF technique and RISA genetic method. Additionally, sequencing of the genes coding for 16S rRNA, isolated from seven strains exhibiting various phenotypic characteristics (Table 1, bolded strains) confirmed their phylogenetic affiliation to SDSE subspecies. All obtained sequences showed over 99 % similarity to SDSE sequences previously deposited in Genbank. Sequences of genes coding for 16S rRNA obtained from six human isolates were also deposited in Genbank under accession numbers KR092084, KR559932, KR559933, KR559934, KR559935, and KR559936, whereas one from animal strain isolated from a dog was deposited in KT232322.

### Antimicrobial Susceptibility

Antibiotic susceptibility profiles of human and animal isolates were roughly similar. The analyzed strains were generally susceptible to β-lactams; however, two human isolates were resistant to penicillin while one animal isolate was resistant to cefotaxime. Moreover, a substantial number of them were resistant to tetracycline, clindamycin, and erythromycin. Three strains showed induced-type MLS_B_ multiresistance (one animal and two human isolates). Among the strains isolated from humans, three were resistant both to vancomycin and linezolid, whereas other three were resistant to tigecycline. Detailed antimicrobial susceptibility results are presented in Table 2.

**Table 2:** Antimicrobial susceptibility profiles of human and animal SDSE isolates

Antibiotic	Penicillin (%)	Erythromycin (%)	Clindamycin (%)	Ofloxacin (%)	Levofloxacin (%)	Vancomycin (%)	Tetracycline (%)	Tigecycline (%)	Linezolid (%)	Cefotaxime (%)
Human SDSE isolates (*N* = 30)										
Susceptible	28 (93 )	17 (57 )	18 (60 )	27 (90 )	29 (97 )	27 (90 )	11 (37 )	27 (90 )	27 (90 )	30 (100 )
Intermediate	0 (0 )	3 (10 )	1 (3 )	3 (10 )	0 (0 )	0 (0 )	5 (16 )	0 (0 )	0 (0 )	0 (0 )
Resistant	2 (7 )	10 (33 )	11 (37 )	0 (0 )	1 (3 )	3 (10 )	14 (47 )	3 (10 )	3 (10 )	0 (0 )
Animal SDSE isolates (*N* = 6)										
Susceptible	6 (100 )	4 (67 )	4 (67 )	5 (83 )	6 (100 )	6 (100 )	2 (33 )	6 (100 )	6 (100 )	5 (83 )
Intermediate	0 (0 )	0 (0 )	0 (0 )	0 (0 )	0 (0 )	0 (0 )	3 (50 )	0 (0 )	0 (0 )	0 (0 )
Resistant	0 (0 )	2 (33 )	2 (33 )	1 (17 )	0 (0 )	0 (0 )	1 (17 )	0 (0 )	0 (0 )	1 (17 )

## Discussion

The first attempt to delineate the division criteria between *S. dysgalactiae* subspecies were undertaken by Vandamme et al. [22] in 1996. They were based on strain isolation source. According to this criterion, Lancefield C or G human isolates were classified to SDSE, whereas all strains isolated from animals were classified to SDSD. This classification reflected neither phenotypic nor genetic diversity of *S. dysgalactiae* species. Only 2 years later, in 1998, the division criteria were updated by Vieira et al. [23]. According to them, β-hemolytic Lancefield group C, G, or L strains were classified to SDSE, whereas nonhemolytic or α-hemolytic Lancefield group C strains were classified to SDSD. The latter division criteria have been used up to the present. However, many exceptions to these rules appear in literature, e.g., SDSE strains with Lancefield group A surface antigens [2, 20]. Moreover, not every SDSE strain is β-hemolytic on sheep blood agar [7]. Because of these ambiguities, some researchers continue to take into account the source of isolation as a division criterion. Therefore, animal infections are being assigned to SDSD strains, whereas human infections are being assigned to SDSE. Despite the fact that the etiological factor of the majority of human infections reported in literature is SDSE [17, 21] and of animal infections—SDSD [1, 10, 18], some cases contradicting this rule were reported, e.g., human infections caused by SDSD [14] and animal infections caused by SDSE [15].

The results of identification of the group of 36 isolates presented in this study question the subspecies division criteria described both by Vandamme et al. [22] and by Vieira et al. [23].

The conducted research suggests that the *S. dysgalactiae* hemolysis type on blood agar should not be longer considered a criterion in subspecies differentiation, at least in analyzed group of strains. Despite the fact that classification to Lancefield groups (C or G) confirms the SDSE characterization described by Vieira et al. [23], it is not an explicit and sufficient criterion. The same C-polysaccharide type (Lancefield group C) is also described as a feature of SDSD strains.

The presented results demonstrate that *S. dysgalactiae* strains isolated from clinical cases in animals might be identified as SDSE by means of currently available methods and their identification according to previously described criteria seems to be questionable.

Currently used *S. dysgalactiae* subspecies division criteria, described by Vieira et al., were established in the 1990s, so nearly 20 years ago. Therefore, currently available techniques allowing to analyze the relationship between strains much more precisely should be introduced. There is a possibility that in described period such changes in strains within *S. dysgalactiae* species occurred which obscure the differences between subspecies noted previously. Such a phenomenon is particularly apparent in bacteria which cross the animal-to-human interspecies barrier. The outline of evolutionary changes during this process was presented in 2007 in *Nature* by Wolfe et al. [25]. An adaptation to a new host is divided into several stages which sometimes might last unexpectedly short. One of the elements of this process, which has been already proven for other streptococci species [12], is horizontal gene transfer (HGT) from related species already adapted to human host. Horizontal gene transfer between *S. pyogenes* and *S. dysgalactiae* [8, 13] has already been recorded. It might lead to the emergence of a new, dangerous pathogen for human with the ability to be transferred via companion animals. We believe that presented results from relatively small group of strains should initiate the discussion about subspecies division criteria re-evaluation.

Antimicrobial susceptibility profiles of human and animal SDSE isolates presented in this study show that multiresistant strains emerge among human isolates. Some of them are also resistant to relatively new antibiotics which additionally enhance the threat.

Due to an increasing genetic variability and phenotypic variability of *S. dysgalactiae* strains isolated both from humans and animals as a result, the division into subspecies established many years ago seems to be questionable, especially when the division criteria are so underspecified. At this moment also, reflections about pan-genome and the general problem of bacterial species definition [11] might be taken into consideration. Although, if the division into two subspecies remains, which in the light of the presented data might be clinically negligible, the division into subspecies should base also on genetic analyses by means of DNA sequences unique for the subspecies (DNA fingerprint), including RISA method presented in this study as well as 16S rRNA genes sequencing. The latter method is also the basis for a phylogenetic relationship tree published in the latest issue of Bergey’s manual of systematic bacteriology [24].
